# A Novel Multi-Objective Binary Chimp Optimization Algorithm for Optimal Feature Selection: Application of Deep-Learning-Based Approaches for SAR Image Classification

**DOI:** 10.3390/s23031180

**Published:** 2023-01-19

**Authors:** Fatemeh Sadeghi, Ata Larijani, Omid Rostami, Diego Martín, Parisa Hajirahimi

**Affiliations:** 1 ETSI de Telecomunicación, Universidad Politécnica de Madrid, Av. Complutense 30, 28040 Madrid, Spain; 2 Spears School of Business, Oklahoma State University, Stillwater, OK 74077, USA; 3 Department of Industrial Engineering, University of Houston, Houston, TX 77204, USA; 4 Department of Business Administration, Boston University, 233 Bay State Road, Boston, MA 02215, USA

**Keywords:** feature selection, POLSAR image classification, improved chimp optimization algorithm, deep convolutional neural network

## Abstract

Removing redundant features and improving classifier performance necessitates the use of meta-heuristic and deep learning (DL) algorithms in feature selection and classification problems. With the maturity of DL tools, many data-driven polarimetric synthetic aperture radar (POLSAR) representation models have been suggested, most of which are based on deep convolutional neural networks (DCNNs). In this paper, we propose a hybrid approach of a new multi-objective binary chimp optimization algorithm (MOBChOA) and DCNN for optimal feature selection. We implemented the proposed method to classify POLSAR images from San Francisco, USA. To do so, we first performed the necessary preprocessing, including speckle reduction, radiometric calibration, and feature extraction. After that, we implemented the proposed MOBChOA for optimal feature selection. Finally, we trained the fully connected DCNN to classify the pixels into specific land-cover labels. We evaluated the performance of the proposed MOBChOA-DCNN in comparison with nine competitive methods. Our experimental results with the POLSAR image datasets show that the proposed architecture had a great performance for different important optimization parameters. The proposed MOBChOA-DCNN provided fewer features (27) and the highest overall accuracy. The overall accuracy values of MOBChOA-DCNN on the training and validation datasets were 96.89% and 96.13%, respectively, which were the best results. The overall accuracy of SVM was 89.30%, which was the worst result. The results of the proposed MOBChOA on two real-world benchmark problems were also better than the results with the other methods. Furthermore, it was shown that the MOBChOA-DCNN performed better than methods from previous studies.

## 1. Introduction

Land-cover and land-use classification are important parts of SAR image application [1,2,3]. According to the past studies, land surfaces can be more accurately classified from POLSAR image datasets. SAR image classification has become an important research issue since these images from RADARSAT-2, ALOS PALSAR, Terra SAR-X, and ENVISAT ASAR have been made available [4]. POLSAR images provide important information about the structures of objects. SAR images are widely used in land-cover classification due to their ability to monitor objects’ structures [5].

POLSAR images play a vital role in land-cover management due to their unique capabilities, including image acquisition in day and night and a weather data acquisition system [2]. Recent advancements in increasing the spatial resolution of POLSAR images provide a considerable chance to obtain more detailed information about the Earth’s surface. However, obtaining high-resolution images requires robust algorithms and classifiers for classification problems [6].

The presence of many features in POLSAR images creates challenges such as high computational times and irrelevant features for applications, which may adversely affect learning tools in both regression and classification [3]. Therefore, to solve these possible challenges and produce accurate results in POLSAR image classification problems, it becomes critical to use a feature selection method (for dimensionality reduction) [2]. Hence, feature selection methods have been studied in many published academic papers. However, most of these methods select features manually based on proposed classifiers. Thus, these methods have a limited ability to accurately select optimal features [7]. Currently, feature selection is still a challenge for POLSAR classification [8].

Feature selection methods are classified into three models: filter-based, embedded, and wrapper methods. Today, meta-heuristic algorithms are one of the most popular wrapper methods for feature selection problems [3]. Meta-heuristic algorithms have become very popular in engineering problems [9,10,11,12,13,14,15,16]. As the complicacy of engineering problems increases, the need for new meta-heuristics becomes obvious more than before. The reasons for this request are simple structures and concepts, derivation-free mechanisms, local optimal avoidance, flexibility, and simple and effective hardware implementation [12]. For this reason, for this paper, a new meta-heuristic algorithm called MOBChOA was used to select the optimal features.

In general, land-cover classification requires powerful algorithms in both the feature selection and classification processes. Classifiers can be broadly divided into two categories: machine learning (ML) and statistical clustering [17,18,19,20]. A well-known statistical classifier is the Wishart classifier, a pixel-based maximum-likelihood classifier based on the complex Wishart distribution of the polarimetric coherency matrix [2]. Moreover, numerous ML methods have been applied to POLSAR image classification, including deep learning (DL), neural networks (NNs), support vector machines (SVMs), and decision trees. However, the most effective model for classifying POLSAR images is not clear. Since 2006, DL has become a popular topic in the ML world [21]. DL models are superior to traditional ML models due to data availability and system processing power developments [22]. Additionally, reducing the computation time and increasing the convergence curve have increased the popularity of these methods. For this reason, for this paper, a deep convolutional neural network (DCNN) was used for land-cover classification.

In the real world, many problems have an inherent binary space, such as feature selection. Moreover, continuous problems can be changed into discrete problems using binary variables [9,10,11,12]. In addition, the no free lunch (NFL) theorem holds that there is no binary meta-heuristic that can appropriately solve all discrete problems [12]. Therefore, the development of new binary algorithms is required to solve discrete problems. Hence, in this study, a new binary version of ChOA is proposed with a new transfer function.

### 1.1. Related Works

SAR is a new remote sensing technique that operates in the microwave frequency range, where it provides low- to high-resolution images of the Earth’s surface using reflected wave signals [23]. Generally, a SAR system operates within the electromagnetic spectrum from 0.3 GHz up to 40 GHz with a side-looking geometry, where a transmitted wave is perpendicular to the direction of the flight of the system [24]. The most important benefits of the SAR system are the ability to work in all weather conditions (smoke, fog, clouds, rain, and day/night), backscatter sensitivity to ground and object features, coherent imaging capability, and the ability to transmit/receive polarized radar waves [25]. Hence, these features are very beneficial for optical and spectral remote sensing systems.

In many remote sensing problems, the scatterers in a volume may have some residual orientation correlation due to the natural structure (branches in a tree canopy, for example) or due to agriculture (oriented corn stalks, for example) [26]. The propagation of radar signals through such a volume can no longer be assumed to be scalar. In this case, the volume has two special polarizations. If there is some mismatch between the radar coordinates and the medium’s special modes, then a very complicated situation arises where the polarization of the incident field changes as a function of the distance into the volume [27].

There are also two major types of configurations we can face in POLSAR images: that of a well-oriented district whose entropy and HV backscattering are low and that of an oriented district with a high HV signal and high entropy, especially when estimated spatially. Disorientation in urban areas is not only followed by an addition to the cross-pol signal but also an increase in entropy. Disorientation leads to random mixing of the mechanisms and makes it impossible or at least very difficult to correct the effect of orientation on the double-bounce mechanism. Even if we are able to highlight the presence of this effect, other involved mechanisms remain mixed in the resolution cell. This leads to the common misclassification results, even with orientation effects [27].

Kajimoto and Susaki [26] proposed an algorithm that robustly extracts urban areas from POLSAR imag41es. Polarization orientation angle (POA) is utilized in the proposed algorithm. A measure of the POA randomness between neighboring pixels is used to discriminate between urban areas with nearly homogeneous POAs and mountain areas with randomly distributed POAs. Experimental results showed that POA-based categorization and the utilization of POA randomness contribute to improving classification accuracy. Without the use of POA randomness, approximately 50% of mountain areas were misclassified as urban areas. Conversely, the addition of POA randomness succeeded in avoiding such a misclassification.

Many widely used SAR image classification algorithms rely on the combination of hand-designed features and machine learning classifiers, which still experience many issues that remain to be resolved and overcome, including the fuzzy confusion of speckle noise, optimized feature representation, widespread applicability, etc. To mitigate some of the issues and to improve the pattern recognition of high-resolution SAR images, Sun et al. [28] developed a ConvCRF model combined with a superpixel boundary constraint. An optimizing strategy using a superpixel boundary constraint in the inference iterations more efficiently preserves structural details. The experimental results demonstrated that the proposed method provides competitive advantages over other widely used models. Zhou et al. [29] used supervised DCNN for POLSAR image classification. With two cascaded convolutional layers, the designed DCNN can automatically learn hierarchical polarimetric spatial features from data. The classification result of the San Francisco case shows that slanted built-up areas, which are conventionally mixed with vegetated areas in polarimetric feature space, can now be successfully distinguished after taking spatial features into account. 

Geng et al. [30] proposed a multi-scale deep feature learning network with bilateral filtering (MDFLN-BF) for SAR image classification, which aims to extract discriminative features and reduce the requirement of labeled samples. MDFLN was also proposed to extract features from SAR images on multiple scales, where the SAR images are stratified into different scales. Experiments showed that the proposed MDFLN-BF is able to yield superior results compared to other related deep networks. Shimoni et al. [31] developed a logistic regression (LR) as a ‘feature-level fusion’ and a neural network (NN) method (for higher-level fusion) to classify PolSAR and polarimetric interferometry (PolInSAR) images. For comparison, a support vector machine (SVM) was also applied. The results of [31] showed that, for both the NN and SVM algorithms, the overall accuracy for each of the fused sets was better than the accuracy for the separate feature sets. Moreover, the fused features from different SAR frequencies were complementary and adequate for land-cover classification. PolInSAR was complementary to PolSAR information, and both were essential for producing accurate land-cover classification. 

Zhang et al. [32] proposed a DL-based unsupervised forest height estimation method based on the synergy of the PolInSAR and light detection and ranging (LiDAR) datasets. Unlike traditional PolInSAR-based methods, the proposed method reformulated the forest height inversion as a pan-sharpening process between the low-resolution LiDAR height and the high-resolution PolSAR and PolInSAR features. UAVSAR PolInSAR and LVIS LiDAR data collected over tropical and boreal forest sites were used for experiments. The experimental results show that the proposed method performed well compared to other traditional methods. Biondi [33] used an improvement of the PolSAR decomposition scheme that permits a more accurate classification. This interferometric polarimetric SAR multi-chromatic analysis (MCA-PolInSAR) method permits the efficient separation of oriented buildings from vegetation, yielding considerably improved results in which oriented urban areas are recognized, from volume scattering, as double-bounce objects. The results also showed a considerable improvement in the robustness of the classification as well as the definition and precision. 

In many studies, the combination of meta-heuristic and ML algorithms has been used in order to select the optimal features and improve the classification accuracy of SAR images [2,3,4,5,6,7,8]. Rostami and Kaveh [3] used a hybrid biogeography-based optimization support vector machine (HBBOSVM) to classify POLSAR images. In the proposed HBBO, the combination of an onlooker bee and a migration operator was used. Then, SVM was applied for land-cover classification. According to the results, the HBBOSVM had better performance than other algorithms in terms of the convergence trend, overall accuracy, and the kappa coefficient. Salehi et al. [3] proposed an integration of multi-objective GA, SVM, and an ANN classifier to find the optimal features in order to improve the accuracy of classification using POLSAR images. The aim of that paper [3] was to minimize the error of classification and the number of selected features. The experimental results of Salehi et al. [3] showed that the proposed model outperformed the single-objective approaches tested against it while saving computational complexity. Finally, their proposed model [3] had a better performance than the SVM and the Wishart classifier.

In general, the majority of the abovementioned studies have proven that the integration of meta-heuristic algorithms and ML tools will achieve better accuracy than traditional feature selection. Hence, in this paper, a combined approach of MOBChOA and CNN methods was used for land-cover classification.

### 1.2. Paper Contributions

According to the mentioned drawbacks and weaknesses of the feature selection methods in the literature, the contributions of this paper are summarized as follows:

- We propose a novel multi-objective binary meta-heuristic algorithm named MOBChOA, in which new concepts such as a transfer function were introduced to better the exploration and exploitation abilities of the ChOA.
- We combined a meta-heuristic algorithm and DL tools to achieve better SAR image classification accuracy.
- By using the proposed deep MOBChOA-CNN architecture, we propose an accurate model for land-cover classification.

### 1.3. Paper Organization

The rest of this paper is organized as follows: Section 2 describes the methodology of the paper, including the dataset, data preprocessing, and the proposed MOBChOA algorithm. Section 3 presents performance evaluations of the MOBChOA-DCNN algorithm for land-cover classification and benchmark problems in comparison with nine competitive algorithms, and finally, we present our conclusions for this paper in Section 4.

## 2. Methodology

**Figure 1:**
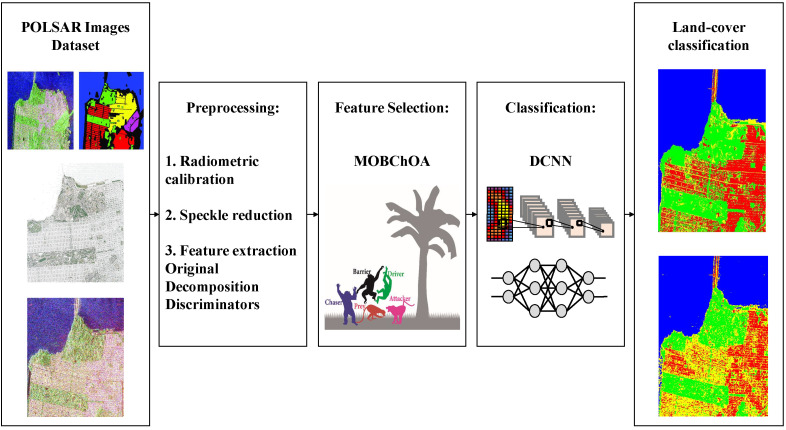
The flowchart of the research method.

In this paper, a DL approach was used as a supervised classification method. Figure 1 shows the research method’s flowchart, which consisted of four major steps. The first step involved performing the necessary preprocessing, which included radiometric calibration, speckle-noise reduction, and feature extraction from POLSAR images as well as generating test and training samples. In the second phase, the proposed MOBChOA was evaluated using two real-world benchmark problems. Then, the proposed MOBChOA-DCNN approach was implemented to select the optimal features. In the third step, the DCNN architecture was applied for land-cover classification (with optimal features selected from MOBChOA). Finally, the classification results were generated as a labeled map.

### 2.1. Dataset

**Figure 2:**
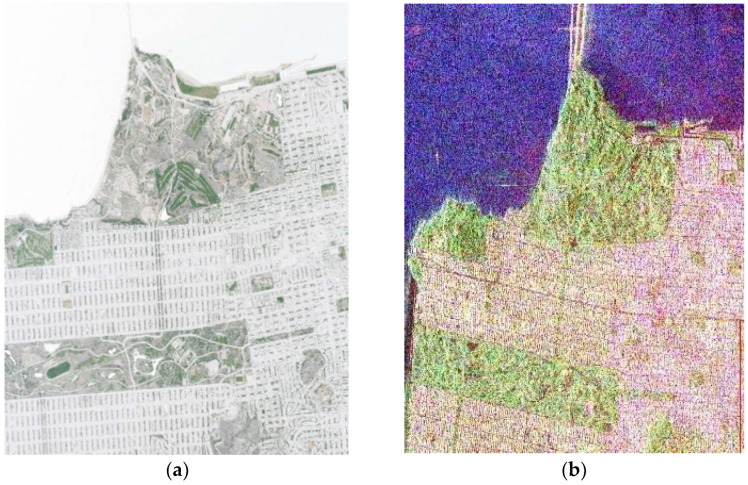
Study area. (**a**) High-resolution image; (**b**) Pauli RGB image.

This paper used the POLSAR images of RADARSAT 2 in the C-band from San Francisco, USA. Figure 2 shows a high-resolution image and a Pauli RGB image of the study area. 

These datasets were collected at 28 and 29.8° incidence angles for the near and far ranges, respectively. Moreover, the approximate spatial resolutions of these orientation were 11.1 and 10.5 m. The San Francisco dataset is one of the most widely used images in SAR image classification in the last decade [2] and includes both man-made and natural objects [2]. The POLSAR image of RADARSAT 2 was a single-look complex (SLC) image. We produced ground truth samples using a high-resolution image, a google earth image, a Pauli RGB image, and a 2006 national land-cover database (NLCD 2006). The pixel size of this dataset was 800 × 1400, which consisted of four main classes: ocean (in blue), vegetation (in green), building (in red), and road (in yellow). Artificial-intelligence-based algorithms must be contextualized in this context. Table 1 indicates the number of testing and training samples collected from high-resolution images from the Pauli RGB image and the 2006 national land-cover database.

### 2.2. Data Preprocessing

This section included performing the necessary preprocessing, which included radiometric calibration, speckle-noise reduction, and feature extraction from POLSAR images. Speckle is known as noise on POLSAR images. Speckle affects the amount of energy recorded due to the interaction between the transmitted and received waves. This salt-and-pepper noise reduces the homogeneity of regions on the POLSAR images [2]. In this paper, we used an improved Lee filter with a window size of 5 × 5 because this method protects edges, linear objects, and texture information.

Radiometric calibration enabled the conversion of the pixel values in the SAR image from being qualitatively representative of the biased backscatter signal to being quantitatively representative of the RCS and the backscatter coefficient, respectively, for the cases of point and extended targets. In this paper, three main methods were used for the radiometric calibration of SAR images:

1. Topography correction: a digital elevation model (DEM) was used to adjust image values because the geometry of the surface affects the pixel values of the image.
2. Converting image values to decibels (dBs): we converted the different units (sigma, beta, and different formats) to dBs using logarithmic transformations.
3. Filtering: a series of functions and special filtering were used.

Generally, POLSAR images have features related to surface roughness and the physical and appearance details of objects on the earth. There are three main types of features to display that are extracted from POLSAR images. These features are divided into original features, discriminators, and decomposition features [2,3,4,5,6,7]. Altogether, 105 features were extracted from the POLSAR image according to [2]. Table 2 shows 101 features that were extracted from the SAR image. 

In this paper, before sampling, various features were extracted using the feature extraction equations of POLSAR images (see [2,34]). In fact, these equations were utilized to convert SLC images into features. The second step was sampling. In this phase, we took the sample from the images and converted it to a datasheet format (vector-matrix). In the datasheet file, the rows were samples and the columns were features. Before entering the data into the proposed DCNN, preprocessing was required. Since the ranges of the feature values were different, the values of all features were normalized between zero and one.

### 2.3. Meta-Heuristic Optimization Algorithms

Concurrently with the new era of information technology, a large number of optimization problems are emerging in different fields such as computer vision, bioinformatics, big data analytics, the Internet of things, etc. [6]. The majority of the real-world optimization problems are NP-hard in nature and cannot be decoded in a polynomial time domain [3]. Rather than giving up, the investigators thought to use possible meta-heuristic algorithms that can find a feasible solution in the given time. Meta-heuristic algorithms can be applied to almost all optimization problems, as they use the optimizer known as the black box [35,36,37]. In designing the meta-heuristic algorithms, two contradictory criteria were considered: exploration in the search space and the exploitation of the best solutions. In exploration, unsearched areas are visited to ensure that all areas of the search space are searched uniformly. Potential areas are explored more fully in exploitation to find a better solution. 

**Figure 3:**
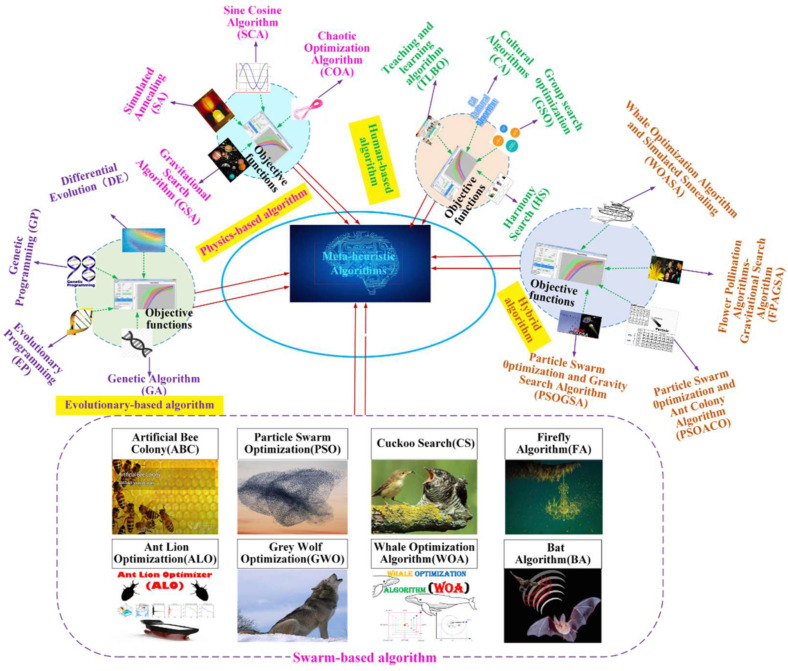
The main categories of meta-heuristic algorithms.

Figure 3 shows the main categories of meta-heuristic algorithms: evolutionary-based algorithms, swarm-based algorithms, physics-based algorithms, human-based algorithms, and hybrid algorithms. Since a few decades ago, a few nature-inspired meta-heuristic algorithms, such as simulated annealing (SA), differential evolution (DE), the genetic algorithm (GA), ant colony optimization (ACO), and particle swarm optimization (PSO) have been introduced and used for optimization problems. Afterward, many studies concentrated on the modification of these algorithms for new applications. Other researchers tried to introduce new meta-heuristic algorithms by taking inspiration from nature. Some newer algorithms such as the chimp optimization algorithm (ChOA), the crystal structure algorithm (CryStAl), red fox optimization (RFO), the honey badger algorithm (HBA), and the gannet optimization algorithm (GOA) are the results of such efforts [35,36,37]. In this paper, a new binary version of ChOA is proposed for the feature selection problem. 

### 2.4. Proposed Algorithm (MOBChOA)

**Figure 4:**
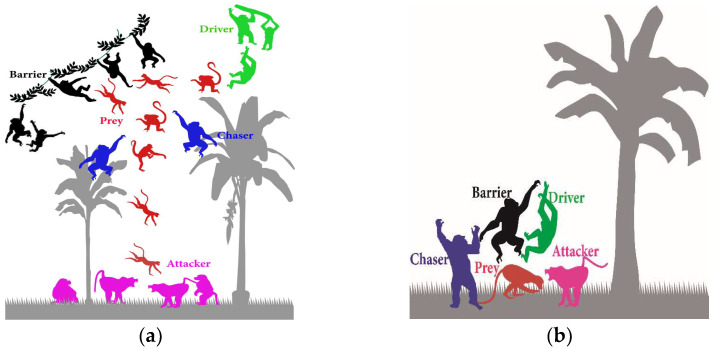
Hunting process in ChOA. (**a**) First phase: exploration, which consists of blocking, driving, and chasing the prey; (**b**) Second phase: exploitation, which consists of attacking the prey.

ChOA was introduced by Khishe and Mosavi in 2020 [12]. ChOA was inspired by chimpanzees’ ability to think individually in group hunting and their sexual motivations. This algorithm divides hunting into four main phases: driving, blocking, chasing, and attacking. ChOA is initialized by generating a random number of chimps. These chimps are then randomly classified into four groups: barrier, attacker, driver, and chaser. Driver chimps follow the prey without trying to reach it. Barrier chimps place themselves in a tree to create a barrier in the prey’s progress. Chaser chimps move quickly to catch the prey. Attacker chimps prognosticate the breakout path of the prey to force it back towards the chasers. Figure 4 indicates the two main phases of the hunting process.

Driving and chasing the prey are formulated as Equations (1)–(5).

(1) $$d=\left|c.X_{prey}\;\left(t\right)-m.X_{chimp}\;\left(t\right)\right|$$

(2) $$X_{chimp}\;\left(t+1\right)=X_{prey}\;\left(t\right)-a.d$$

(3) $$a=2.f.\;r_{1}-f$$

(4) $$c=2.\;r_{2}$$

**Figure 5:**
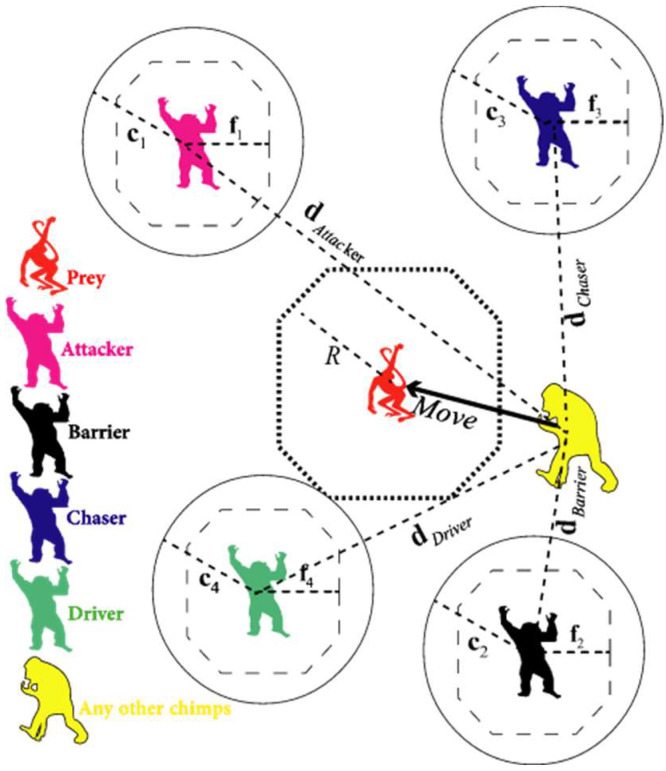
Position updating in ChOA.

(5) m=Chaotic_value

where $X_{prey}$ is the prey position vector; $X_{chimp}$ denotes the chimp position vector; t presents the current iteration; a, c, and m are the coefficient vectors; f is the dynamic vector ∈[0, 2.5], $r_{1}\;\mathrm{and}\;r_{2}$ are the random vectors ∈[0, 1]; and m denotes a chaotic vector. Chimpanzees first find the position of the prey in the hunting phase using blocker, driver, and chaser chimps. The position of the prey is then calculated by barrier, attacker, chaser, and driver chimps, and other chimpanzees update their positions via the prey. These phases are formulated as Equations (6)–(8).

(6) $$\{\begin{array}{c} d_{Attacher}=\left|c_{1}.X_{Attacher}-m_{1}.X\right|\\ d_{Barrier}=\left|c_{2}.X_{Barrier}-m_{2}.X\right|\\ d_{Chaser}=\left|c_{3}.X_{Chaser}-m_{3}.X\right|\\ \;d_{Driver}=\left|c_{4}.X_{Driver}-m_{4}.X\right| \end{array}$$

(7) $$\{\begin{array}{c} X_{1}=X_{Attacher}-a_{1}\left(d_{Attacher}\right)\\ X_{2}=X_{Barrier}-a_{2}\left(d_{Barrier}\right)\\ X_{3}=X_{Chaser}-a_{3}\left(d_{Chaser}\right)\\ \;X_{4}=X_{Driver}-a_{4}\left(d_{Driver}\right) \end{array}$$

(8) $$X\;\left(t+1\right)=\frac{X_{1}+X_{2}+X_{3}+X_{4}}{4}$$

where $X_{Attacher}$ presents the best search agent, $X_{Barrier}$ is the second-best search agent, $X_{Chaser}$ denotes the third-best search agent, $X_{Driver}$ is the fourth-best search agent, and X (t+1) is the updated position of each chimp (Figure 5).

Finally, all chimpanzees attack their prey after hunting the prey to achieve sexual motivation regardless of their duties. Sexual motivations are formulated using chaotic maps (Equation (9)):

(9) $$X_{chimp}\left(t+1\right)=\{\begin{array}{c} X_{prey}\left(t\right)-a.d\;\;\;\;\;\;\;\;\;\;\;\;\;\;\;\;\;\;if\;\;\;\;\mu <0.5\;\\ Chaotic\_value\;\;\;\;\;\;\;\;\;\;\;\;\;\;\;if\;\;\;\mu \;\geq \;0.5\;\; \end{array}$$

where μ is the random number ∈ [0, 1]. In the continuous version of ChOA, chimpanzees constantly change their position at any point in space. In discrete problems, the solutions are restricted to binary values. Operators of binary meta-heuristic methods can only move to the nearer and farther corners of the hypercube by shifting 0 to 1 and 1 to 0. Therefore, in the BChOA design, the position update equation should be modified. For this purpose, a transfer function is needed to map the continuous space to a discrete space. The transfer function characterizes the probability of changing the position vector elements from 0 to 1 and vice versa. Thus, this transfer function compels chimpanzees to move in discrete space. In this section, a new method for updating the positions of chimpanzees is introduced. In the proposed BChOA, the position updating equation is formulated as Equation (10). Here, a sigmoid function (transfer function) is used as Equation (11):

(10) $$X_{d}^{t+1}=\{\begin{array}{c} 1\;\;\;\;\;\;\;\;\;\;\;\;\;\;\;\;if\;sigmoid\;\left(\frac{X_{1}+X_{2}+X_{3}+X_{4}}{4}\right)\geq R\;\;\;\;\;\;\\ 0\;\;\;\;\;\;\;\;\;\;\;\;\;\;\;\;\mathrm{otherwise}\;\;\;\;\;\;\;\;\;\;\;\;\;\;\;\;\;\;\;\;\;\;\;\;\;\;\;\;\;\;\;\;\;\;\;\;\;\;\;\;\;\;\;\;\;\; \end{array}$$

(11) $$Sigmoid\;\left(x\right)=\frac{1}{1+e^{-14\left(x-0.45\right)}}$$

where $X_{d}^{t+1}$ is the updated binary position at iteration t; R is a random number ∈[0, 1]; Sigmoid (x)  is the S-shaped functions, $X_{1},\;X_{2},X_{3},\;\mathrm{and}\;X_{4}$, which denote the chimpanzees movements towards the attacker, barrier, chaser, and driver chimps, respectively.

**Figure 6:**
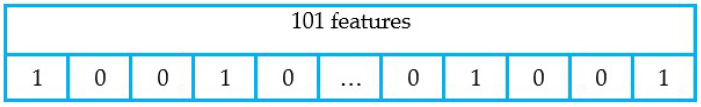
Definition of a chimp in the MOBChOA algorithm.

Figure 6 shows the formulation of the feature selection problem as a chimpanzee definition in the MOBChOA algorithm. Since there are 101 features in the SAR dataset, the chimp has an array of 101 features. In Figure 6, a feature value of 1 shows that this feature is used for classification.

In this study, two objective functions were used for land-cover classification: maximum overall accuracy (OA) and the minimum number of features. The weighted sum method was used to integrate the two objective functions. Therefore, the fitness function was formulated as Equation (12):

(12) $$Fitness\;Function\;\left(i\right)=\alpha .OA\left(i\right)+\left(1-\alpha \right).\log_{10}\frac{N}{n\left(i\right)}$$

where  Objective Function (i) is the fitness function of the ith  chimp, OA(i) is the overall accuracy of the ith  chimp, N=101 features, and n(i) is the number of features selected in the ith  chimp. Furthermore α is the weight parameter, which is considered to be 0.92. The calibration of α was set by a trial-and-error method. To find the best value for each of the parameters, the other variables were kept fixed and the objective function was implemented with different values of parameters. Moreover, the value of the fitness function was considered as the main criterion for the measurement and calibration of the parameters. According to the trial-and-error method as well as previous studies [3], the best value of α was equal to 0.92.

## 3. Experimental Results

This section is divided into two subsections. In the first subsection, the performance of the proposed MOBChOA algorithm on two real-world benchmark optimization problems was evaluated in comparison with four competitive algorithms, including the capuchin search algorithm (CapSA) [38], black widow optimization (BWO) [39], the grey wolf optimizer (GWO) algorithm [40], and biogeography-based optimization (BBO) [41]. In the second subsection, to evaluate the performance of MOBChOA-DCNN for land-cover classification, three neural network architectures called long short-term memory (LSTM), the radial basis function neural network (RBFNN), the multiple-layer perceptron neural network (MLPNN) and two classical architectures called random forest (RF) and SVM were used. All architectures were coded in MATLAB, and Table 3 indicates the optimal parameters of the algorithms.

### 3.1. Real-World Benchmark Optimization Problems

Two real optimization problems were used to evaluate the performance of MOBChOA. The problems considered in this section assessed the proposed MOBChOA from different perspectives. Due to the complexity of real optimization problems, solving them can be a challenge for proposed algorithms [42].

#### 3.1.1. Design of Tension Spring

**Figure 7:**
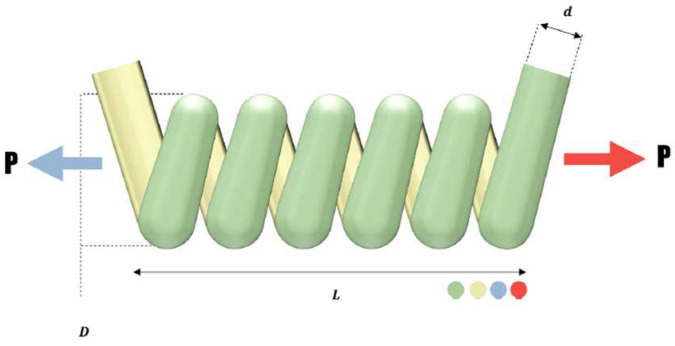
Tension spring problem.

The design of a tension spring is a complex problem that was used in this paper. A schematic view of the problem is shown in Figure 7 and can be formulated as Equations (13)–(18) [43]:

(13) $$f\left(X\right)=(x_{3}+2)x_{2}x_{1}^{2}$$

subjected to

(14) $$g_{1}\left(X\right)=1-\frac{x_{3}x_{2}^{3}}{71,785x_{1}^{4}}\leq 0$$

(15) $$g_{2}\left(X\right)=\frac{4x_{2}^{2}-x_{1}x_{2}}{12,566\left(x_{2}x_{1}^{3}-x_{1}^{4}\right)}+\frac{1}{5108x_{1}^{2}}-1\leq 0$$

(16) $$g_{3}\left(X\right)=1-\frac{140.45x_{1}}{x_{2}^{2}x_{3}}\leq 0$$

(17) $$g_{4}\left(X\right)=\frac{x_{1}+x_{2}}{1.5}-1\leq 0$$

(18) $$0.05\leq x_{1}\leq 2.00,\;0.25\leq x_{2}\leq 1.30,\;2\leq x_{3}\leq 15$$

where:

$x_{1}$ = wire diameter (d).

$x_{2}$ = mean coil diameter (D).

$x_{3}$ = the number of active coils (N).

**Figure 8:**
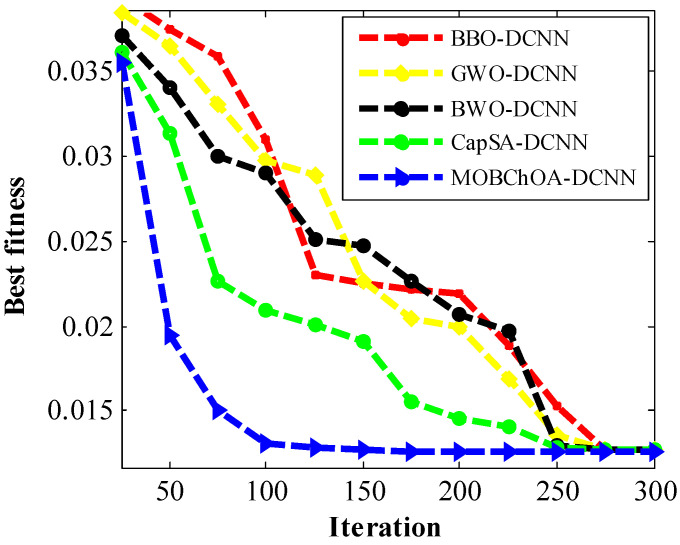
The convergence trends of algorithms for the spring system problem.

Table 4 shows the implementation results of the MOBChOA and other algorithms for this problem. As can be seen, MOBChOA found the best value for the fitness function. The best solution that was found for the problem was 0.0126652. The MOBChOA convergence trend for this problem is shown in Figure 8, which shows that the convergence curve of MOBChOA was faster than those of the other algorithms.

#### 3.1.2. Three-Bar Truss Design Problem

**Figure 9:**
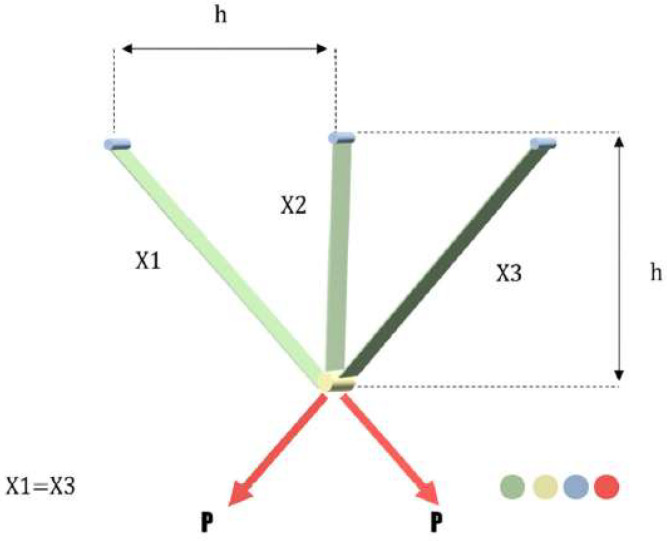
A schematic view of the three-bar truss design problem.

The three-bar truss problem is a widely used real-world engineering optimization problem. The three-bar truss problem intends to find the minimum weight of a three-bar truss. A schematic view of the problem is shown in Figure 9 and can be formulated as Equations (19)–(23) [43]:

(19) $$f\left(X\right)=\left(2\sqrt{2x_{1}}+x_{2}\right)\times l$$

subjected to

(20) $$g_{1}\left(X\right)=\frac{\sqrt{2}x_{1}+x_{2}}{\sqrt{2}\;x_{1}^{2}+2x_{1}x_{2}}P-\sigma \leq 0$$

(21) $$g_{2}\left(X\right)=\frac{x_{2}}{\sqrt{2}\;x_{1}^{2}+2x_{1}x_{2}}P-\sigma \leq 0$$

(22) $$g_{3}\left(X\right)=\frac{1}{\sqrt{2}\;x_{2}+x_{1}}P-\sigma \leq 0$$

(23) $$0\leq x_{1},\;\;x_{2}\leq 1$$

where:

$x_{1}$ = $A_{1}$.

$x_{2}$ = $A_{2}$.

$x_{3}$ = $x_{1}=A_{3}$.

l=100 cm.

$P=2\;\mathrm{KN}/{\mathrm{cm}}^{2}$.

$\sigma =2\;\mathrm{KN}/{\mathrm{cm}}^{2}$.

**Figure 10:**
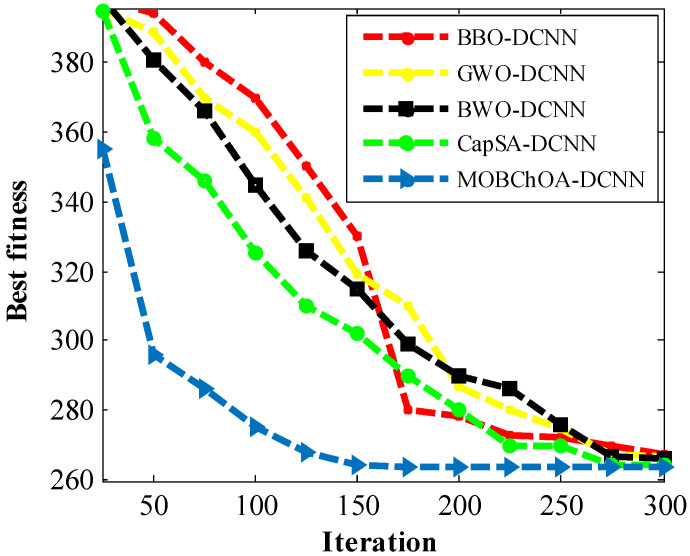
The convergence trends of the algorithms for the spring system problem.

Table 5 shows the comparison of the MOBChOA results with the results from the other algorithms. As can be seen, MOBChOA found the best value for the fitness function. The best solution that was found for the problem was 263.895843. According to the results, MOBChOA had the best standard deviation. Figure 10 indicates the convergence curves of MOBChOA and the other algorithms. The convergence curve of MOBChOA was faster than those of the other algorithms.

### 3.2. SAR Image Classification

**Figure 11:**
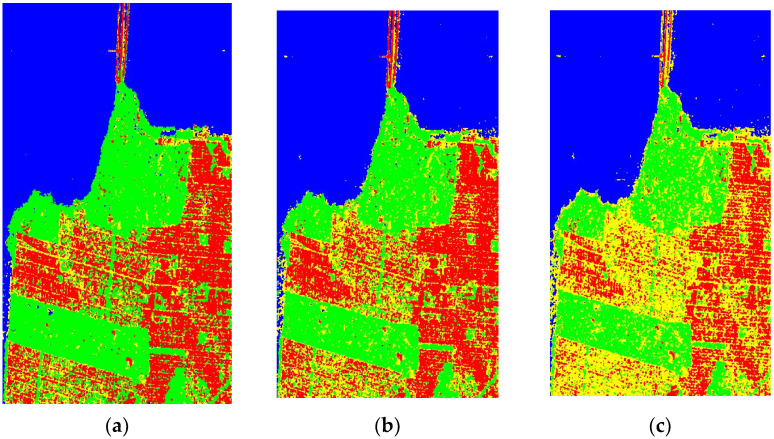

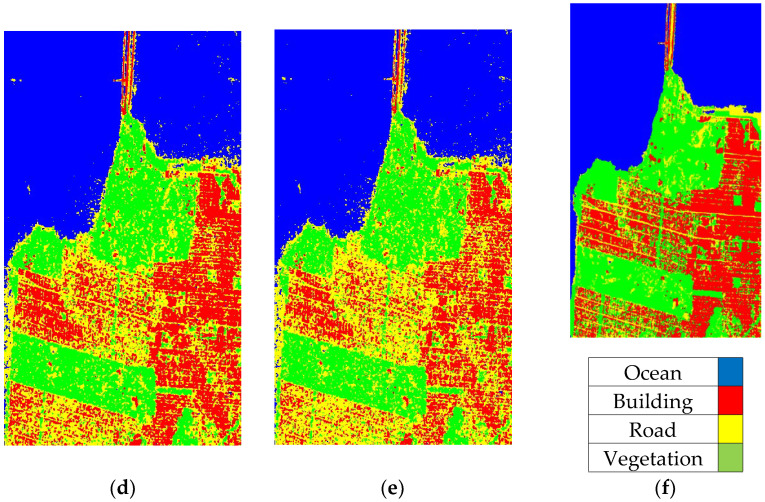
Classified images of architectures. (**a**) MOBChOA-DCNN; (**b**) CapSA-DCNN; (**c**) BWO-DCNN; (**d**) GWO-DCNN; (**e**) BBO-DCNN; (**f**) Ground truth image.

Figure 11 shows the images classified by MOBChOA-DCNN, CapSA-DCNN, BWO-DCNN, GWO-DCNN, and BBO-DCNN. On the left side of the study area, the results indicate a low-density urban area, and on the right side of the study area the results indicate a high-density urban area. According to Figure 11, the classified images based on the Pauli RGB image, the high-resolution image, and the ground truth samples were accurately identified.

**Figure 12:**
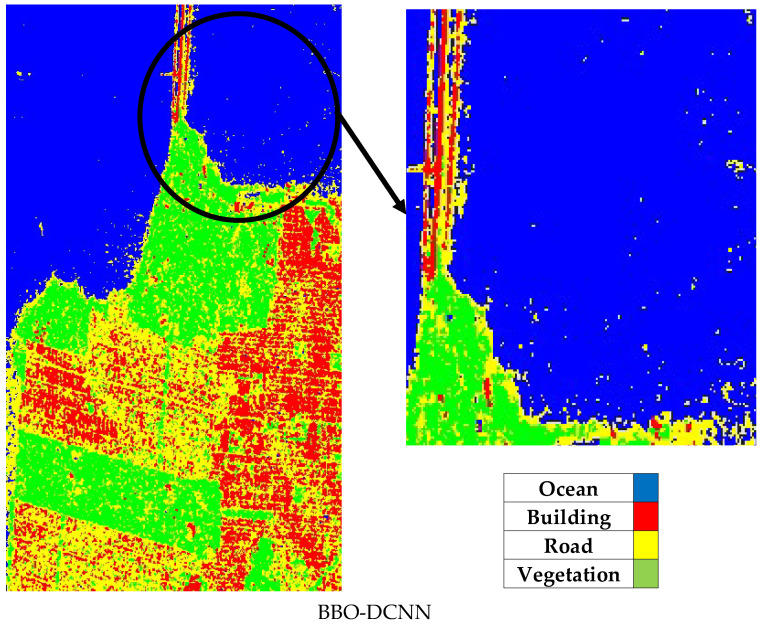
Misclassified ocean pixels.

**Figure 13:**
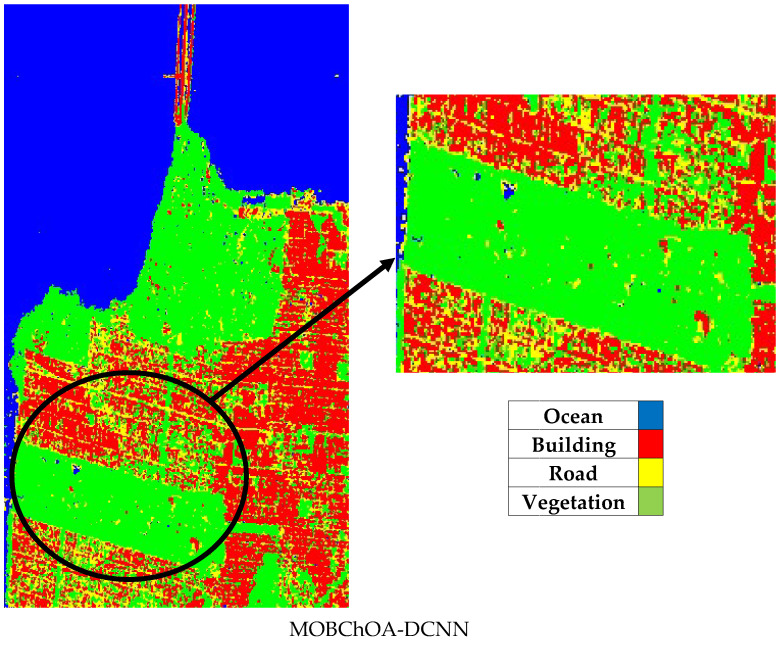
Some flat surfaces inside the vegetation.

As shown in the classified images, MOBChOA-DCNN accurately detected the building and ocean classes. In Figure 11b–e, some ocean pixels were misclassified due to the appearance of waves and speckle at the sea surface. Figure 12 shows these misclassified pixels. However, in MOBChOA-DCNN, the ocean class was well identified. According to the ground truth samples and classified images (Figure 11), there were some flat surfaces inside the vegetation that were not roads (in the center-left of the classified images). Figure 13 shows these flat surfaces within the vegetation. The road pixels also had a flat surface. The flat surface scattering mechanism in POLSAR images is double-bounce. For this reason, those flat surfaces were considered double-bounce scattering (road class), and MOBChOA-DCNN detects these areas accurately.

For the validation of classified images, sensitivity, overall accuracy, and specificity metrics were used to compare the efficiency of the hybrid architectures. These criteria can be calculated using Equations (24)–(26) [44]:

(24) $$Sensitivity=\frac{TP}{TP+FN}$$

(25) $$Specificity=\frac{TN}{TN+FP}$$

(26) $$Accuracy=\frac{TP+TN}{TP+FN+FP+TN}$$

where TP = true positive, FN = false negative, TN = true negative, and FP = false positive. Table 6 shows the specificity, sensitivity, and overall accuracy of architectures for land-cover classification. As can be seen, MOBChOA-DCNN showed the highest efficiency on the training and validation datasets. MOBChOA-DCNN achieved 96.89% and 96.13% accuracy values in the testing and training datasets, respectively. Moreover, the results of the DL architectures trained by meta-heuristics were better than the LSTM, RBFNN, MLPNN, RF, and SVM architectures. As shown in Table 6, the “Average RunTime” of MOBChOA-DCNN was shorter than those of the other algorithms for land-cover image classification.

**Figure 14:**
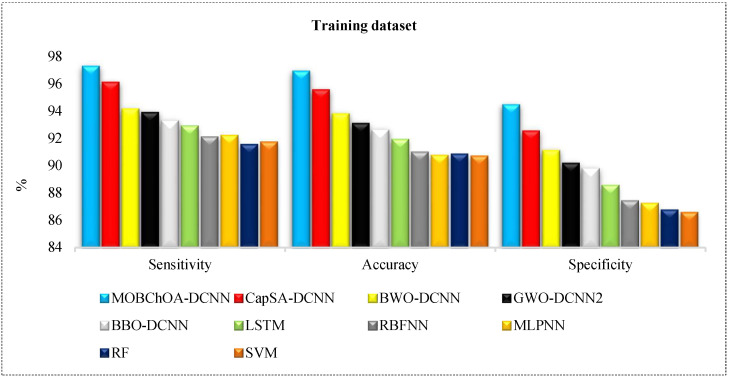
Comparison of architectures in training dataset.

**Figure 15:**
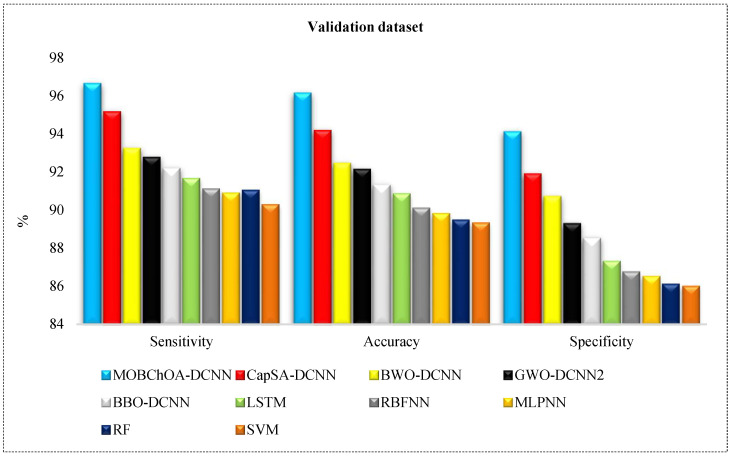
Comparison of architectures in validation dataset.

Figure 14 and Figure 15 provide comparisons of the algorithms according to these metrics. According to these figures, the ranking of the algorithms was MOBChOA-DCNN, CapSA-DCNN, BWO-DCNN, GWO-DCNN, and BBO-DCNN, respectively. The results of MOBChOA-DCNN on the testing and training datasets showed that the proposed feature selection method performed well because the specificity, accuracy, and sensitivity of MOBChOA-DCNN were highly stable.

Table 7 provides a comparison of the architectures according to mean square error (MSE) criteria. MOBChOA-DCNN had a smaller MSE than the other architectures. The MSE function can be calculated using Equation (27), where n is the total number of samples, $y_{i}$ is the system output, and $d_{i}$ shows the desired value:

(27) $$MSE=\frac{1}{n}\;\overset{n}{\underset{i=1}{\sum }}{\left(y_{i}-d_{i}\right)}^{2}\;$$

**Figure 16:**
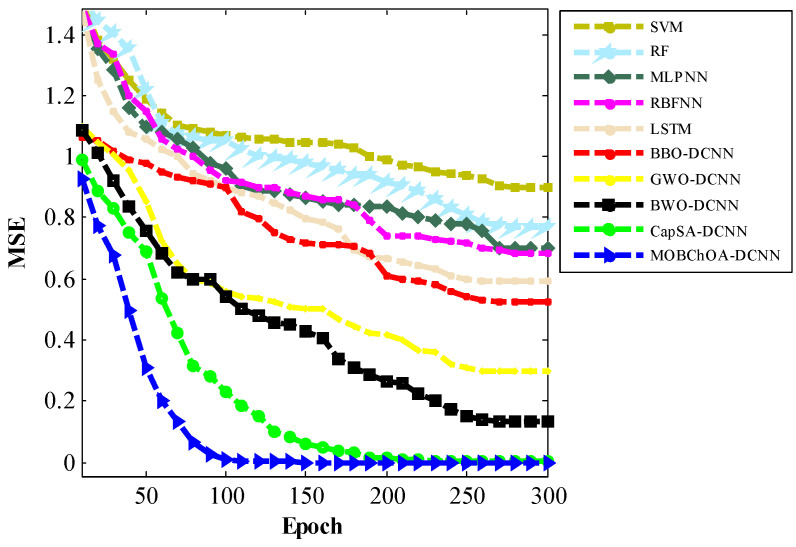
The convergence curves of the architectures for the MSE criterion.

In this paper, a new transfer function was proposed to map continuous space to discrete space. The transfer function characterizes the probability of changing the position vector elements from 0 to 1 and vice versa. The transfer function compels chimpanzees to move in discrete space. Therefore, it helps the algorithm to avoid being trapped in local minimums. According to the results, the proposed MOBChOA-DCNN method was useful for image classification. Figure 16 shows the convergence trends of the architectures. As shown in this figure, the MOBChOA-DCNN and CapCA-DCNN architectures converged faster than the others. Moreover, MOBChOA-DCNN achieved high stability and a high convergence speed in fewer epochs. The reason for MOBChOA’s superiority is the existence of three operators: (a) exploration, which consists of blocking, driving, and chasing the prey; (b) exploitation, which consists of attacking the prey; and (c) a new transfer function to map the continuous space to discrete space.

Table 8 compares the results of this paper and previous research (that was prepared by Salehi et al. [2]). As can be seen, MOBChOA-DCNN had better accuracy than the other algorithms. The ranking of the algorithms was MOBChOA-DCNN, CapSA-DCNN, GASVM [3], BWO-DCNN, GWO-DCNN, BBO-DCNN, SVM [2], GA-MLP [2], and Wishart [2], respectively. In the research by Salehi et al. [2], the overall accuracy of Wishart [2] using nine features was 75.33%. On the other hand, the overall accuracy of SVM [2] using 105 features (all features) was 90.40%. By the comparison of classifiers, when using meta-heuristic algorithms, classification accuracy was improved and redundant features were also removed. In general, the proposed MOBChOA-DCNN provided fewer features and the highest overall accuracy.

## 4. Conclusions and Future Works

We proposed a hybrid approach of meta-heuristic algorithms and DL methods for land-cover classification. To do so, we first performed the necessary preprocessing, including speckle reduction, radiometric calibration, and feature extraction. After that, we proposed a novel algorithm named MOBChOA to improve the exploitation and exploration of the ChOA for optimal feature selection. Finally, we trained the fully connected DCNN to classify POLSAR images from San Francisco, USA.

In this paper, the performance of the MOBChOA on two benchmark problems was evaluated in comparison with four competitive algorithms, including CapSA, BWO, GWO, and BBO. Moreover, to evaluate the performance of MOBChOA-DCNN for land-cover classification, five competitive architectures called LSTM, RBFNN, MLPNN, RF, and SVM were utilized. The experimental results on the POLSAR image dataset showed that the proposed model (MOBChOA-DCNN) could achieve better accuracy and a better convergence rate while reducing the number of features. Furthermore, the performance compared to the previous research showed that the proposed MOBChOA-DCNN model outperformed the state-of-the-art algorithms. When using meta-heuristic algorithms, classification accuracy was improved and redundant features were removed.

Like most meta-heuristics, there are many operators in the proposed MOBChOA. Therefore, modeling these operators for real-world problems can be a challenge. The accurate setting of the initial parameters of MOBChOA is a limitation, and special methods should be used. Another challenge in real-world problems is the complexity of MOBChOA. Calculating the fitness functions of all solutions and choosing the best solution causes computational complexity.

Several research directions can be recommended for future works. Modified variants of MOBChOA may be extended to tackle various multi-objective, discrete, and real-world optimization problems. Moreover, a few selective parameters and thresholds in MOBChOA’s equations have not been optimally fine-tuned, providing a path for further work. Hybrid algorithms will improve the operators. In addition, tackling problems in different fields (i.e., neural networks, image processing, scheduling, data mining, big data, smart homes, industry, etc.) could be a valuable and beneficial contribution.

## Figures and Tables

**Table 1:** The number of testing and training samples.

Class	# Pixels	# Training	# Testing
1. Ocean	422,347	6083	416,264
2. Road	121,926	867	121,059
3. Building	269,186	1628	267,558
4. Vegetation	306,541	1086	305,455
Total	1,120,000 (800 × 1400)	9664	1,110,336

**Table 2:** Features extracted from the SAR image.

Feature	Description	Symbol	Number
Original features	Covariance matrix	C	9
	Coherency matrix	T	9
	Scattering matrix	S	4
Decomposition features	Krogager	krog	3
	Huynen	h	9
	Barnes	B	9
	Cloude	Cl	9
	Holm	Hol	9
	Vanzyl	V	3
	Cloude–Pottier	H, A, alpha, lambda, gamma, delta, asym, anisotropy, HA, (1-H)A, H(1-A), (1-H)(1-A), RVI	19
	Freeman–Durden	Fd	3
	Yamaguchi	Y	4
	Touzi	Toz	4
Discriminator features	Correlation coefficients	CC	4
	Polarized intensity	Pi	1
	Degree of polarization	D	1
	Span	S	1

**Table 3:** The optimal parameter settings of the algorithms.

Algorithm	Parameter	Value
MOBChOA	a	[−1, 1]
	f	Linearly decreased from 2 to 0
	Population size	150
	Iteration	300
CapSA	Velocity control constants	1.00
	Inertia parameter	0.64
	Balance and elasticity factors	0.73, 9
	Population size	150
	Iteration	300
BWO	Procreation rate (PP)	0.66
	Mutation rate (PM)	0.25
	Cannibalism rate (CR)	0.48
	Population size	150
	Iteration	300
GWO	C	0.7
	A	0.3
	α	Linearly decreased from 2 to 0
	Population size	150
	Iteration	300
BBO	The probability range for migrating	[0, 1]
	Maximum emigration (I) and immigration (E)	1
	Elitism percent	6%
	Mutation rate	0.08
	Population size	150
	Iteration	300

**Table 4:** Comparison of the results of the algorithms for spring system design.

Algorithm	Best Fitness	Mean Fitness	Standard Deviation	Iteration
MOBChOA	1.26652 × 10^−2^	1.28705 × 10^−2^	0.0000356	300
CapSA	1.26849 × 10^−2^	1.38564 × 10^−2^	0.0028536	300
BWO	1.26986 × 10^−2^	1.44263 × 10^−2^	0.0325691	300
GWO	1.27083 × 10^−2^	1.46896 × 10^−2^	0.6523281	300
BBO	1.27176 × 10^−2^	1.87563 × 10^−2^	1.2360894	300

**Table 5:** Comparison of the results of the algorithms for the three-bar truss design problem.

Algorithm	Best Fitness	Mean Fitness	Standard Deviation	Iteration
MOBChOA	263.895843	265.896523	0.0000209	300
CapSA	264.325698	272.745632	0.0125634	300
BWO	265.796325	276.745263	0.1396589	300
GWO	266.896352	278.749236	0.7058932	300
BBO	267.105962	279.785632	2.8963254	300

**Table 6:** Algorithm results for the validation of classified images.

Deep Architectures	Training Dataset			Validation Dataset			Run Time
	Sensitivity	Specificity	Accuracy	Sensitivity	Specificity	Accuracy	
MOBChOA-DCNN	97.25%	94.43%	96.89%	96.63%	94.09%	96.13%	832 s
CapSA-DCNN	96.08%	92.51%	95.53%	95.14%	91.88%	94.16%	986 s
BWO-DCNN	94.12%	91.08%	93.76%	93.21%	90.69%	92.43%	1056 s
GWO-DCNN	93.86%	90.14%	93.06%	92.75%	89.26%	92.12%	1008 s
BBO-DCNN	93.24%	89.73%	92.58%	92.16%	88.49%	91.29%	1161 s
LSTM	92.86%	88.52%	91.89%	91.64%	87.29%	90.83%	1351 s
RBFNN	92.07%	87.39%	90.96%	91.09%	86.73%	90.08%	1269 s
MLPNN	92.18%	87.19%	90.72%	90.86%	86.49%	89.79%	1412 s
RF	91.52%	86.72%	90.81%	90.19%	86.08%	89.45%	1096 s
SVM	91.69%	86.53%	90.67%	90.26%	85.98%	89.30%	1196 s

**Table 7:** Comparison of the algorithms according to MSE.

Proposed Architectures	MSE	
	Training Dataset	Validation Dataset
MOBChOA-DCNN	0.00019	0.00093
CapSA-DCNN	0.00386	0.05856
BWO-DCNN	0.13201	0.29632
GWO-DCNN	0.29872	0.49632
BBO-DCNN	0.52361	0.97526
LSTM	0.59368	1.10395
RBFNN	0.68395	1.22368
MLPNN	0.70196	1.31856
RF	0.76589	1.38596
SVM	0.89652	1.42698

**Table 8:** Comparison of the results of this paper and previous research [2].

Algorithm	Overall Accuracy (%)	Number of Features
Wishart [2]	75.32	9
SVM [2]	90.40	105
GA-MLP [2]	87.38	56
GA-SVM [2]	93.65	37
MOBChOA-DCNN (ours)	96.13	27
CapSA-DCNN (ours)	94.16	31
BWO-DCNN (ours)	92.43	33
GWO-DCNN (ours)	92.12	32
BBO-DCNN (ours)	91.29	35

## Data Availability

The datasets generated during and/or analyzed during the current study are available from the corresponding author on reasonable request.
